# Feasibility of a multigroup Boltzmann–Fokker–Planck solution for electron beam dose calculations

**DOI:** 10.1038/s41598-023-27376-y

**Published:** 2023-01-24

**Authors:** Ahmed Naceur, Alain Hébert, Paul Romano, Benoit Forget, Cornelia Chilian, Jean-François Carrier

**Affiliations:** 1 Department of Engineering Physics, Nuclear Engineering Institute, École Polytechnique, Montréal, H3T1J4 Canada; 2 Department of Mechanical Engineering, Nuclear Engineering Institute, École Polytechnique, Montréal, H3T1J4 Canada; 3 Computational Science Division, Argonne National Laboratory, Lemont, IL60439 USA; 4 Department of Nuclear Science and Engineering, Massachusetts Institute of Technology, Cambridge, MA02139 USA; 5 CRCHUM, Centre hospitalier de l’Université de Montréal, Montréal, H2L4M1 Canada; 6 Department of Physics, Université de Montréal, Montréal, H3T1J4 Canada

**Keywords:** Oncology, Engineering, Mathematics and computing, Physics, Applied physics, Nuclear physics, Statistical physics, thermodynamics and nonlinear dynamics

## Abstract

Legacy nuclear-reactor Boltzmann solvers start clinical deployment as an alternative to Monte Carlo (MC) codes and Fermi–Eyges semiemprical models in radiation oncology treatment planning. Today’s certified clinical solvers are limited to photon beams. In this paper, ELECTR, a state-of-the-art multigroup electron cross sections generation module in NJOY is presented and validated against Lockwood’s calorimetric measurements, EGS-nrc and GEANT-4 for 1–20 MeV unidirectional electron beams. The nuclear-reactor DRAGON-5 solver is upgraded to access the library and solve the Boltzmann–Fokker–Planck (BFP) equation. A variety of heterogeneous radiotherapy and radiosurgery phantom configurations were used for validation purpose. Case studies include a thorax benchmark, that of a typical breast Intra-Operative Radiotherapy and a high-heterogeneity patient-like benchmark. For all beams, $100\%$ of the water voxels satisfied the American Association of Physicists in Medicine accuracy criterion for a BFP-MC dose error below $2\%$. At least, $97.0\%$ of adipose, muscle, bone, lung, tumor and breast voxels satisfied the $2\%$ criterion. The average BFP-MC relative error was about $0.56\%$ for all voxels, beams and materials combined. By irradiating homogeneous slabs from $Z=1$ (hydrogen) to $Z=99$ (einsteinium), we reported performance and defects of the CEPXS mode [US. Sandia National Lab., SAND-89-1685] in ELECTR for the entire periodic table. For all Lockwood’s benchmarks, NJOY-DRAGON dose predictions are within the experimental data precision for $98\%$ of voxels.

## Introduction

The NJOY nuclear data processing system is widely used for processing pointwise and multigroup neutron and photon cross sections from Evaluated Nuclear Data Files (ENDF)^1^. The current restriction to neutral particle-induced evaluations limits the scope of the system’s application to fission reactor design, licensing and safety analysis, stockpile stewardship modeling, criticality safety benchmarking, radiation shielding and nuclear waste management^2,3,4^.

*The need.* Light charged particle transport is required in, among others, ultra-scaled electronic devices^5^ (e.g., silicon microelectronic devices^6^), low-pressure fusion plasma control^7^, gas-discharge plasma^8^, accelerator beam transport (e.g., (e$^-$, e$^+$) colliders)^9,10^, beam–beam interactions^5^, radiation oncology and medical physics^11–13^. The use of electron transport codes/models is already pervasive in radiation oncology daily clinical practice workflow. To avoid the stochastic nature of a Monte Carlo (MC) calculation—known to be highly accurate but computationally-expensive and time-consuming—medical physicists have resorted to the so-called *kernel semi-empirical models* (SEM). Modified MC algorithms, e.g., modifying electron transport, limiting tracking of low-probability events or implementing voxel-based transport methods^14^ and those based on variance reduction techniques^15^ exist in some clinical routines^16,17^ and will not be discussed here.

Point kernel^18^, pencil beam^19–21^, collapsed cone convolution^22^ and convolution/superposition^23,24^ are the models typically deployed in clinical treatment planning systems (TPS). The main assumptions stem from the use of Fermi–Eyges small-angle scattering theory^25,26^ for radiative transfer, which states that (i) charged particle multiple scattering involves only very small variations in the direction of propagation, (ii) electrons have a small angle of flight, i.e., their trajectories are contained within a cone, preventing straying from production sites, and (iii) all electrons at depth *x* have a predetermined energy *E*(*x*). Consequently, such approximations incorrectly equate particle path length to its depth, ignore straggling effects, catastrophic energy loss and large-angle deviation. In 1981, Hogstrom et al.^27^ proposed the first adaptation of this theory for electron beams (uncoupled transport). Because of the depth dependence of the predetermined kernel, the model can only account for stratified heterogeneities^28^. The latter is being approximated by a diffusing kernels’ rescaling. This pencil beam model was generalized 13 years later by Gustafsson^29^ and Ulmer^30^ for photon beams. From the outset, these studies have reported considerable failures ranging from cases of simple heterogeneities to complex configurations. Correction factors and SEM improvements—e.g., Jette and Bielajew second-order multiple-scattering theory^31^, Storchi and Huizenga angular stopping power ingredient^32^, Bruinvis et al. straggling model^33^, Shiu and Hogstrom redefinition algorithm^34^, Yu et al. multiray model^35^, Ahnesjö et al. (photon beams)^21^ and Knoos et al. (electron beams)^36^ partial heterogeneity corrections, Ulmer et al. lateral scaling^37^, or Tillikainen et al.^38^ buildown models—were necessary but have not prevented typical errors of $22\%$ (following a density perturbation)^39^ or $40\%$ (near heterogeneities)^40^ from reoccurring. Hensel et al.^28^ explain that the problem is that the Fermi–Eyges multiple scattering hypothesis is known to be true in astrophysics, but it cannot be true for human tissues. In other words, even if elastic Mott and inelastic Møller and Bhabha scatterings are forward peaked, the cumulative effect of multiple scattering results in a considerable angle change for which the Fermi–Eyges theory was not developed for. Clinicians are aware of these limitations^41–43^.

*The effort.* In the last few years, two equivalent efforts in two different fields (medical and nuclear) attempted to tackle kernel SEM and MC drawbacks by developing deterministic transport capabilities for charged particles. The nuclear effort has chosen to build on the existing neutronic theory, multigroup formalism and nuclear reactor Boltzmann solvers, while the medical effort made the choice to start over from scratch, laying out the theory and developing rough drafts of homemade algorithms.

Larsen^44,45^ showed that; (i) neglecting large-angle scattering leads, systematically, to significant errors, (ii) the Fermi–Eyges and the Fokker–Planck equations are at least 10 orders of magnitude from the BFP accuracy. Jette^46^ demonstrated that a *Gaussian multiple-scattering equation*, based on the BFP equation, leads to a more accurate scattering kernel than the pencil beam one. Larsen et al.^47^ showed, with low-order moments and a Gaussian imposed flux, that a coarse numerical resolution of the BFP equation in infinite water slab results in dose profiles closer to MC than Fermi–Eyges theory. Tervo et al.^48,49^ proposed a 1D finite-element inverse planning BFP algorithm, coupling photon and electron transport in biological tissues while omitting bremsstrahlung and pair production. Hensel et al.^28^ claimed a first presentation of a complete coupled Boltzmann equation for photon radiotherapy in heterogeneous tissues. All these studies are either irreproducible, built from scratch, ignore some interactions, far from the $2\%$ accuracy criterion^14,50^, or based on unevaluated cross sections. Thus, they do not meet typical requirements for quality, completeness, robustness, reproducibility and automation^51^.

The first reproducible results, with quality assurance, were proposed by Gifford et al.^52^ and Vassiliev et al.^50^ following the introduction of the Los Alamos Attila SN solver, respectively, for Fletcher Suit Delclos and Rogers and Mohan benchmarks (2006), prostate and head-and-neck tumor photon beam treatment plans (2008). A $3\%$ (pointwise relative difference) dose agreement was observed for $99\%$ of the voxels in build-up regions, near heterogeneities, and at the beam penumbra. In 2010, Vassiliev et al.^53^ proposed Acuros, an optimized rewrite of the general purpose Attila code, and showed that a $2\%$ dose agreement for $99.9\%$ of voxels in breast photon treatment planning is possible. The qualification, validation and certification of Acuros has therefore grown from year to year in radiation oncology. In 2011, Bush et al.^54^ showed that Acuros reduced typical Anisotropic Analytical Algorithm (AAA) $10.2\%$ and $17.5\%$ clinical errors, respectively, in lung and low lung density, to $2.0\%$ and $2.9\%$. In 2012, Hoffmann et al.^55^ qualified Acuros with ionization chamber detectors for homogeneous (within $1\%$) and heterogeneous tissues (within $2\%$) and again demonstrated its superiority over AAA clinical algorithm, especially in bone and lung tissues. From 2013 to 2022, similar performance was reported for Intensity-Modulated Radiotherapy (IMRT), rapidArc of nasopharyngeal carcinoma^56^, Stereotactic Body Radiation Therapy (SBRT)^57^, rapidArc planning for non-small-cell lung tumor, stereotactic and conventional lung volumetric modulated arc therapy^58^, metallic hip implant interference^59^, high-dose-rate brachytherapy treatment planning^60^, volumetric modulated arc therapy plans^61^, lung SBRT treated during Deep Inspiration Breath Hold (DIBH)^62^, patient clinical treatment plans (e.g., lung/3D conformal, lung/IMRT, head and neck/VMAT, cervix/IMRT and rectum/VMAT)^63^, stereotactic ablative body radiotherapy^64^, hepatocellular carcinoma stereotactic body radiation therapy and spine anthropomorphic SBRT^65^.

*The state-of-the-art limits.* Five limits are to be mentioned: (1) previous studies only concern photon radiotherapy, (2) Vassiliev et al.^53^ mention that electron radiotherapy is not possible with Acuros due to the lack of an implementation of the Fokker–Planck continuous scattering operator, (3) cross sections are based on the 1989 US. Sandia National Laboratory CEPXS library (SAN89-1685, Version 1.0)^66^, (4) the latter is a coupled electron–photon unevaluated multigroup-Legendre cross section generation code, the only code of its kind, which had a single release (October, 1989) and has never been validated for electron radiotherapy and (5) CEPXS, Acuros and Attila are not available under open source licenses.

*The proposal.* In this paper, we try to tackle these problems. First, we propose and validate ELECTR, a state-of-the-art multigroup electron cross section generation module in NJOY, which can work with ENDF data as well as CEPXS cross sections. A GENDF-formatted library—containing multigroup catastrophic electroatomic cross sections, relaxation cascade production, anisotropic Legendre components of within-group elastic and group-to-group inelastic scattering matrices, radiative and collisional soft stopping powers, multigroup momentum transfer coefficients, energy and charge deposition cross sections, covering [1 keV, 20 MeV] range for elements $Z = 1$ (hydrogen) to $Z = 99$ (einsteinium)—is produced by the ELECTR module. Secondly, we upgrade the MATXSR post-processing module in NJOY to format the latter library in a single stand-alone multigroup dataset that can be accessed by a variety of legacy discrete ordinates and lattice codes. The produced MATXS file is then recovered by the upgraded Dragon-5 BFP discrete ordinate solver^67^, where a high-order diamond differencing (HODD) scheme is used for spatial discretization, with a $P_{\ge 8}$ Legendre expansion for scattering anisotropy and $S_{64}$ angular quadrature. Finally, we proceed with validations against Lockwood’s experimental calorimetric measurements^68^, Egs-nrc, Geant-4 on water slabs, thorax^69^, intraoperative Mobetron electron radiotherapy^70^ and high-heterogeneity benchmarks^41^ as well as typical slabs covering the entire periodic table. This paper is limited to the case of CEPXS feed functions in ELECTR. For validation reasons, only electrons are transported. Bremsstrahlung and fluorescence photons are produced and eliminated at the place of birth.

## Methods

### Boltzmann–Fokker–Planck equation

Let *n* be the electron discrete ordinate direction (${\hat{\Omega }}_{n}$), *g* its energy group, $\vec {r}$ its position, the multigroup form of the $S_{N}$ BFP equation is then given by:

1 $$\begin{aligned} {\hat{\Omega }}_{n}\cdot \vec {\nabla }\psi _{g,n}^e(\vec {r})+\Sigma _{t,g}(\vec {r})\psi _{g,n}^e(\vec {r}) = L^{\text {B}}_{g,n}\psi _{g,n}^e(\vec {r}) + L^{\text {FP}}_{g,n}\psi _{g,n}^e(\vec {r}) + Q_{g,n}^{e,\text {ext.}}(\vec {r}). \end{aligned}$$

$\psi ^e$ designates the electron flux, $\Sigma _t$ the total cross section, $L^{\text {B}}$ the Boltzmann operator, $L^{\text {FP}}$ the Fokker–Planck operator and $Q^{e,\text {ext.}}$ the external electron source. All these quantities are discretized on the phase space ${{\mathscr {D}}}$. According to Fano’s [${1}\,{\textrm{keV}}$, ${100}{\textrm{GeV}}$] classification^71^, the considered interactions are the bremsstrahlung (b), inelastic collision/ionization (c), elastic collision (e) Auger, Coster–Kronig and super Coster–Kroning cascades (a). Although fluorescence cascades are implemented in ELECTR, here we limit our study to pure electron transport. The electron flux satisfies the periodicity condition. Its development in spherical harmonics ($R_{l}^{m}$) is written as:

2 $$\begin{aligned} \psi _{g,n}^e(\vec {r}) = \sum \limits _{l=0}^{L} \sum \limits _{m=-l}^{+l} \psi ^{e}_g(\vec {r}) R_{l}^{m}({\hat{\Omega }}_{n}), \ \forall (\vec {r},g,n)\in {{\mathscr {D}}}. \end{aligned}$$

where *L* refers to the Legendre order. Flux moments $\psi _{l,g}^m(\vec {r})$ can be obtained by integrating Eq. 2 over all incidence directions and normalizing with the complex conjugate $R_{l}^{m^*}$. $L^{\text {B}}$ describes the rate of catastrophic collisions. $L^{\text {B}}$ has a rotation invariant kernel, i.e., its eigenfunctions are the $R_{l}^{m}$ spherical harmonics and the associated eigenvalues are the Legendre scattering cross section coefficients, $\Sigma _{s,l}^x$. *x* designates the electroatomic interaction, i.e., $x\in \{a,b,c,e\}$. Using Eq. 2, decomposing the scattering cross sections into Legendre polynomials, then using Legendre Addition Theorem and finally exploiting the orthogonality of the $R_{l}^{m}$ harmonics, the Boltzmann operator is written as:

3 $$\begin{aligned} L^{\text {B}}_{g,n}\psi _{g,n}^e(\vec {r}) = \sum \limits _{x}^{} \sum \limits _{l=0}^{L} \sum \limits _{m=-l}^{+l} \sum \limits _{g'}^{G} \ \Sigma _{l,g'\rightarrow g}^{x}(\vec {r}) \psi _{l,g'}^{m}(\vec {r}) R_{l}^{m}({\hat{\Omega }}_n) \end{aligned}$$

$L^{\text {FP}}_{g,n}$ operator is obtained by a Taylor polynomial expansion of the Boltzmann $L^{\text {B}}_{g,n}$ operator around small energy-loss and small-angle scattering^72^. Considering only the first-order Taylor expansion, $L^{\text {FP}}_{g,n}$ can be written as the sum of the continuous slowing-down ($L^{\text {FP}_1}_{g}$) and the continuous scattering operator ($L^{\text {FP}_2}_{g,n}$).

4 $$\begin{aligned} L^{\text {FP}}_{g,n}=L^{\text {FP}_1}_{g}\psi _{g,n}^e(\vec {r}) + L^{\text {FP}_2}_{g,n}\psi _{g,n}^e(\vec {r}), \ \ \text {where} \ \ L^{\text {FP}_1}_{g}= \frac{\partial \beta _{g}(\vec {r})}{\partial E_{g}}, \ \ L^{\text {FP}_2}_{g,n}=\frac{1}{2} \alpha _{g}(\vec {r}) \left[ \frac{\partial }{\partial \mu _{n}}\left( 1-\mu _{n}^2\right) \frac{\partial }{\partial \mu _{n}} + \frac{1}{1-\mu _{n}^2} \frac{\partial ^2}{\partial \phi _{n}^2}\right] . \end{aligned}$$

$\beta _{g}$ and $\alpha _{g}$ designate, respectively, the restricted stopping power and the restricted momentum transfer. Restriction refers to soft collisions. Here, the considered source is monoenergetic and unidirectional. If $Q_e(\vec {r})$ is the source intensity and ${{\mathscr {D}}}_{Q}$ its spatial domain:

5 $$\begin{aligned} Q_{g,n}^{e,\text {ext.}}(\vec {r}) = Q_e(\vec {r}) \delta (E-E_g) \delta ({\hat{\Omega }} - {\hat{\Omega }}_n), \ \forall \vec {r} \in D_{Q}. \end{aligned}$$

The system Eqs. 1 and 5 is considered complete after applying the boundary conditions.

### Microscopic electroatomic catastrophic cross sections

Neutronic formalism^73–76^ is used to define electroatomic multigroup Legendre coefficient transfer matrices ($\Sigma _{l}^{x}$) and multigroup total cross sections ($\Sigma _{g}^t$). A Gauss–Lobatto quadrature is used to evaluate all integrals in ELECTR. The nodes and weights are provided up to the 10th order. For a given interaction, *x*, microscopic catastrophic cross sections are given by:

6 $$\begin{aligned} \sigma _{\ell g\rightarrow g'}^x = \frac{\int _g{{\mathscr {F}}}^{x,0}_{\ell g'}(E)\,\phi _\ell (E)\,dE}{\int _g\phi _\ell (E)\,dE}; \quad \sigma _{g}^{x}=\frac{\int _g{\mathscr {F}}^{x,0}(E)\,\phi _0(E)\,dE}{\int _g\phi _0(E)\,dE}. \end{aligned}$$

where *g* and $g'$ refer to the incident and transfer energy groups, respectively. $\phi _\ell$ is a Legendre component of the electron flux’s guess. For both collisional and radiative interactions, a catastrophic event is assumed to be one in which down-scattering happens into non-adjacent energy groups. ${{\mathscr {F}}}(E)$ is a unifying concept of the so-called *feed function*. This concept was introduced by MacFarlane et al.^77,78^ in the NJOY GROUPR and GAMINR modules for neutron- and photon-production cross sections and is adapted here for electron-production cross sections. It alone changes for different data types. For matrices, ${\mathscr {F}}_{lg'}^{x,0}(E_g)$ is the ${\ell }$th Legendre moment of the normalized probability of scattering into secondary energy group $g'$ from initial energy $E_g$. For vectors, ${{\mathscr {F}}}^{x,0}(E_g)$ is the total cross section interaction from the same initial group $E_g$. The general form of the *m*th moment of the ${\ell }$th Legendre order group-to-group feed function (MeV$^{m}$ $\cdot$barns) is given by:

7 $$\begin{aligned} {{\mathscr {F}}}^{\textrm{x},m}_{\ell g'}(E_g) \negthinspace = \negthinspace \int _{-1}^1 \text {d}\mu \,P_\ell ({\hat{\Omega }}\cdot {\hat{\Omega }}') \int _{I_{g'}^{\textrm{x}}} dE' \, \sigma _{\textrm{x}}(E_g\rightarrow E',{\hat{\Omega }}\cdot {\hat{\Omega }}')\,(E')^m. \end{aligned}$$

where ${\hat{\Omega }}$ and ${\hat{\Omega }}'$ stand for the incident and scattered particle directions, respectively. $P_l$ is the Legendre polynomial. $I_{g'}^{\textrm{x}}$ is the electron transfer domain for interaction *x*. The differential scattering cross section (DSC) is given by:

8 $$\begin{aligned} \sigma _{\textrm{x}}(E_g\rightarrow E',{\hat{\Omega }}\cdot {\hat{\Omega }}') = \sigma _{\textrm{x}}(E_g) {\mathscr {P}}_{\textrm{x}}(E_g \rightarrow E') {{\mathscr {D}}}_{\textrm{x}}({\hat{\Omega }}\cdot {\hat{\Omega }}') \end{aligned}$$

${{\mathscr {P}}}_x$ is the microscopic differential energy distribution kernel, while ${{\mathscr {D}}}_x$ refers to the microscopic differential angular distribution kernel (both for the *x* interaction).

*Ionization feed function.* Two electrons emerge from each *k*th atomic subshell ionization by an incident electron of energy $E_g$: the scattered electron and the delta ray (also called recoil electron). By convention, the particle with the lower energy is identified as the delta ray, and the one with the higher energy is the principal scattered electron. The total ionization feed function for group $g'$ is then the sum over all subshells of the *m*th moment of the ${\ell }$th Legendre order for primary electron transferring to group $g'$ (${{\mathscr {F}}}^{k,ee,m}_{\ell g'}(E_g)$) and the *m*th moment of the ${\ell }$th Legendre order for delta ray production and transferring to group $g'$ (${\mathscr {F}}^{k,e\delta ,m}_{\ell g'}(E_g)$), all starting with incident energy $E_g$ impacting on the *k*th subshell:

9 $$\begin{aligned} {{\mathscr {F}}}^{\textrm{c},m}_{\ell g'}(E_g) \negthinspace = \sum _{k} {\mathscr {F}}^{k,ee,m}_{\ell g'}(E_g) + {{\mathscr {F}}}^{k,e\delta ,m}_{\ell g'}(E_g) \end{aligned}$$

By substituting Eq. 7 in 9, the questions then become (i) how ELECTR proceeds for ${\mathscr {P}}_{k,ee/e\delta }$ and ${{\mathscr {D}}}_{k,ee/e\delta }$ distributions (Eq. 8); and (ii) what are the transfer domains $I^{k,ee}_{g'}$ and $I^{k,e\delta }_{g'}$ (Eq. 7) for both scattered and delta electrons. It depends on the operation mode, i.e., if it is an ENDF or a CEPXS feed function mode. Here, the operation “mode” should not be confused with the data “format”. In both cases, the energy and the scattering angle of the primary and secondary electrons are deduced from the conservation laws of the collision kinematics. In either case, it is assumed that no kinetic energy is transferred to the residual atom. For the ENDF mode, three-body collision kinematics are used, namely: the incident particle, the secondary emitted one and the subshell involved. ${{\mathscr {P}}}_{k,e\delta }$ distributions are all provided in the ENDF evaluation as MF=26, MT=534–572 for all materials. The MF and MT values are part of the ENDF-6 format for evaluated nuclear data. The MF value, running from 1 to 99, encodes the class of information (e.g., MF=23: interaction cross section, MF=26: angular distribution). The MT value, running from 1 to 999, encodes the interaction type (e.g., MT=102: neutron capture, MT=527: electron bremsstrahlung). ${{\mathscr {D}}}_{k,ee}$ and ${{\mathscr {D}}}_{k,e\delta }$ are both Dirac delta distributions around the cosine of the scattering angle of the emitted particle. The subshell’s binding energy and ionization total cross section ($\sigma _k(E)$ in Eq. 8) are, respectively, provided in MF=28, MT=534–572 and MF=23, MT=534–572. The transfer domains for the primary and secondary electron are given by:

10 $$\begin{aligned} I_{g'}^{k,ee} = [\text {max}(E_{g'},E/2),\text {min}(E_{g'+1},E-E_{\text {cut}})]\quad \text {and} \quad I_{g'}^{k,e\delta } = [\text {max}(E_{g'},E_{\text {cut}}),\text {min}(E_{g'+1},E/2)], \forall k. \end{aligned}$$

where $E_{\text {cut}}$ is a threshold integration limit chosen to avoid feed functions’ divergence for catastrophic collisions. *E*/2 is the lowest kinetic energy that the principal scattered electron can have. For the CEPXS mode, the ionization is done on a free electron and therefore there is no threshold condition nor any atomic shell or subshell involved. For both the primary and secondary electrons, the DSC (Eq. 8) is that of Møller, for which the angular distributions remain Dirac distributions around the cosine of the scattering angles obtained, in this case, by a two-body kinematics^79^. Unlike in condensed-history MC codes, energy-loss straggling models are not necessary in ELECTR since differential ${\mathscr {P}}_{k,e\delta }$ distributions already account for this effect.

*Bremsstrahlung feed functions.* One electron and one photon emanate from this interaction. Whether for ENDF or CEPXS mode, no angular deflection is allowed for the bremsstrahlung electron. ${{\mathscr {D}}}_{b}$ in Eq. 8 is therefore a Dirac delta distribution around ${\hat{\Omega }}\cdot {\hat{\Omega }}'=1$. The photon, for both ENDF and CEPXS modes, is emitted according to a Sommerfeld angular distribution^80^. The energies of the two particles are obtained by the conservation balance. Eqs. 7 and 8 can be simplified, in this case, as follows:

11 $$\begin{aligned} {{\mathscr {F}}}^{\textrm{b},m}_{\ell g'}(E_g) \negthinspace = {{\mathscr {F}}}^{\textrm{b},m}_{0 g'}(E_g) = \int _{I_{g'}^b} \text {d}E' \ \sigma _{b}(E_g) {{\mathscr {P}}}_b (E_g\rightarrow E_g-E') (E')^m, \quad I_{g'}^b=[\text {max}(E_{g'},E_{\text {min}}),\text {min}(E_{g'+1},E-E_{\text {cut}})]. \end{aligned}$$

$E_{\text {min}}$ is the high frequency limit of bremsstrahlung divergence. It is fixed at 1 keV for both modes. $E_{\text {cut}}$ has the same definition as in Eq. 10. For the ENDF mode, ${{\mathscr {P}}}_b$ and $\sigma _b$ in Eq. 11 are interpolated, respectively, from MF=26, MT=527 and MF=23, MT=527, taking into account bremsstrahlung production in nucleus and Coulomb electric fields. For the CEPXS mode, the distribution is implemented analytically according to Koch and Motz DSC assembly based on relativistic Born approximation with Coulomb correction^81^. Empirical parameters, e.g., Elwert corrections and screening factors, are retrieved from CEPXS Tape9 database^66^. The Koch and Motz DSC also diverges when the energy of the photon approaches that of the incident electron^82^. The same $I_{g'}^b$ domain introduced in Eq. 11 is also used in this mode. Bremsstrahlung production from interaction with atomic electrons’ field is accounted by modifying the Koch and Motz $Z^2$ factor by $Z(Z+1)$.

*Elastic feed function* The nucleus-scattered electron is deviated without change in energy (${\mathscr {P}}_e=\delta _{gg'}$). Equations 7 and 8 are then simplified as follows:

12 $$\begin{aligned} {{\mathscr {F}}}^{\textrm{e}}_{\ell g'}(E_g) \negthinspace = {{\mathscr {F}}}^{\textrm{e},0}_{\ell g}(E_g) \negthinspace = \negthinspace \int _{-1}^1 \text {d}\mu \,P_\ell (\mu ) \sigma _{\textrm{e}}(E_g) D_{\textrm{e}}(\mu ) \end{aligned}$$

For the ENDF mode, $\sigma _{\textrm{e}}(E_g)$ is interpolated from MF=23, MT=525. For *large* angular scattering ($\mu \in [-1.0, 0.999999]$), the $D_{\textrm{e}}$ distribution is interpolated from MF=26, MT=525^83^. For forward scattering ($\mu \in [0.999999,1.0]$), Seltzer’s analytical screened Coulomb DSC^84,85^ is implemented. From Cullen’s EEDL classification^83^, elastic scattering is considered either large or forward-peaked. For all atoms, ENDF angular distributions are provided below and above ${256}\,{\textrm{keV}}$. In the CEPXS mode, the Mott DSC with Molière screening^86^ is used for relativistic energies ($E_g>{256}\,{\textrm{keV}}$), while a Riley DSC^87^ is used for lower energies. For both Mott and Riley distributions, and instead of evaluating the Legendre moments in Eq. 12 with quadratures, the CEPXS mode uses Berger’s semi-analytical approach^88^ based on Goudsmit–Saunderson distribution and Spencer functions^89^. All empirical parameters are retrieved from CEPXS database^66^. The final step in both modes is to modify the *l*th Legendre order of the elastic feed function by an extended transport correction, similar to the one proposed by Bell for neutrons^90^ in nuclear reactor physics. The transport-corrected within-group elastic feed function is given by:

13 $$\begin{aligned} \bar{{{\mathscr {F}}}}^{\textrm{e}}_{\ell g}(E_g) = {{\mathscr {F}}}^{\textrm{e}}_{\ell g'}(E_g) - {{\mathscr {F}}}^{\textrm{e}}_{L g'}(E_g) \end{aligned}$$

where *L* is the maximum Legendre order. As shown by Morel for electrons^91^, such an approximation makes the highly forward-peaked elastic scattering kernel compatible with low-order Legendre expansion and, potentially, a replacement for $L^{\text {FP}_2}$ in Eq. 4.

*Auger relaxation cascade.* In both modes, it is assumed that Auger electrons are emitted isotropically. Let *j* be the transition line, $\eta _{kj}^e$ its relaxation efficiency (i.e., that the $j$th transition produces an Auger electron following the *k*th shell or subshell impact ionization), the $m$th moment of the $l$th Legendre order of Auger production feed function is given by:

14 $$\begin{aligned} {{\mathscr {F}}}^{\textrm{a},m}_{\ell g'}(E_g) \negthinspace = \sum _{k} \sigma _k(E_g) \sum _{j=1}^{N_{\text {tr}}} \eta _{kj}^e \delta _{g'g_j} (e_{kj}^e)^m, \forall l=0. \end{aligned}$$

Higher-order Auger production feed functions are identically zero. $g_j$ is the electron group that contains the $j$th emitted Auger energy, while $e_{kj}^e$ refers to the actual Auger, Coster–Kronig or super Coster–Kronig electron energy. $N_{\text {tr}}$ is the number of possible atomic transitions. For the ENDF mode, transition probabilities and the emitted particle spectra are both interpolated from MF=28, MT=534–572, which describe subshell ionization for the K1 to O5 shells. For the CEPXS mode, only the K, L1, L2, L3 and M shells are involved in relaxation cascades, which results in $N_{\text {tr}}=28$ different transitions after an impact event. Also, for this mode, $e_{kj}^e$ is expressed in terms of the midpoint group energy rather than the line radiation one. Binding energies and probability parameters are stored in Tape9 relaxation quantities^66^. For both modes, the relaxation algorithm does not contribute to the total reaction rate.

*Total macroscopic cross sections.* For all interactions, the total catastrophic cross section can be obtained by summing the group-to-group feed function (Eq. 7) over all incident angles:

15 $$\begin{aligned} {{\mathscr {T}}}_g^{\text {x}} = {{\mathscr {F}}}_{0g'}^{\text {x},0}. \end{aligned}$$

The upper limit of $I_{g'}^{x}$ (Eqs. 15 and 8) is set in such a way that the threshold energy is arbitrarily selected at the interface $E_{g-1}$ between soft and catastrophic collisions. Its lower limit is the lowest energy that the scattered electron could have (e.g., *E*/2 for Møller scattering).

### Microscopic restricted stopping powers

Total stopping powers (MeV$\cdot$barns) are converted in ENDF-6 format from Berger’s evaluation^92^. ELECTR interpolates collisional (${{\mathscr {S}}}^c_g$) and radiative (${{\mathscr {S}}}^b_g$) total stopping powers from the converted MF=23, MT=507 and MF=23, MT=508 sections, respectively. Since Bethe ${{\mathscr {S}}}^c_g$ shows imperfections below ${10}\,{\textrm{keV}}$, due to negligence of deviations from plasmon, inner subshells’ electrons and conduction electrons^93^, ELECTR corrects ${{\mathscr {S}}}^c_g$ below ${10}\,{\textrm{keV}}$ using Lorence’s power-law extrapolation^66^. Catastrophic stopping powers, ${{\mathscr {M}}}^c_g$ and ${{\mathscr {M}}}^b_g$, are taken as the first moment of the total catastrophic cross section. Before being averaged, restricted stopping powers are evaluated at all group boundaries except the last one.

16 $$\begin{aligned} \beta _g^c = {{\mathscr {S}}}^c_g - {{\mathscr {M}}}^c_g = {{\mathscr {S}}}^c_g - {\mathscr {F}}_{0g'}^{c,1} = S_g^c - \int _{E/2}^{E_{g-1}} \text {d}E' (E-E') \sigma _c(E \rightarrow E').\end{aligned}$$

17 $$\begin{aligned} \beta _g^b = {{\mathscr {S}}}^b_g - {{\mathscr {M}}}^b_g = {{\mathscr {S}}}^b_g - {{\mathscr {F}}}_{0g'}^{b,1} = S_g^b - \int _{E_{\text {min}}}^{E_{g-1}} \text {d}E' (E-E') \sigma _{b}(E \rightarrow E'). \end{aligned}$$

where, for ${{\mathscr {M}}}_g$, we followed the state-of-the-art recommendations by integrating Møller (in Eq. 16) and Koch and Motz (in Eq. 17) DSCs. Integrals’ limits are identical to the previous ones, i.e., a non-divergence lower limit and a soft-catastrophic interface upper limit. The total restricted stopping power (Eq. 4) is the sum of Eqs. 16 and 17. In this study, lack of stopping powers below ${1}\,{\textrm{keV}}$ impose a transport cutoff at ${1}\,{\textrm{keV}}$. This cutoff is in line with recommendations made by Salvat to Cullen for EPICS-2017 data^83,94,95^. It is also in compliance with the default cutoff limits of Penelope^96^, Egsnrc^97^ and Fluka^98^.

### Microscopic energy and charge deposition cross sections

Energy deposition cross section (MeV$\cdot$barns) account for the energy deposited in each electron–matter interaction. It can take either a positive (local energy deposition) or a negative (local energy removal) value. The energy transported with the scattered electron or the emitted Auger, Coster–Kronig, or super Coster–Kronig electron are typical examples of negative energy deposition cross sections. This quantity is central for a deterministic dose assessment in when solving the BFP equation. Three phenomena are contributing: inelastic collision, bremsstrahlung and Auger cascades. In the first two cases, one should sum contributions from soft and catastrophic collisions. Inelastic collisional and bremsstrahlung energy deposition cross sections are given by

18 $$\begin{aligned} {{\mathscr {E}}}_{g'}^{c}(E_g)= & {} \sum _k \left[ {{\mathscr {T}}}_{g'}^k(E_g) E_{g'} - \sum _{h=1}^{g'-2} {{\mathscr {F}}}^{k,ee,1}_{0 h}(E_g) + {\mathscr {F}}^{k,e\delta ,1}_{0 h}(E_g) \right] + \beta _{g'}^c({\bar{E}}_{g'}) \end{aligned}$$

19 $$\begin{aligned} {\mathscr {E}}_{g'}^{b}(E_g)= & {} {{\mathscr {T}}}_{g'}^b(E_g) E_{g'} - \sum _{h=1}^{g'-2} {{\mathscr {F}}}^{b,1}_{0 h}(E_g) + \beta _{g'}^b({\bar{E}}_{g'}) \end{aligned}$$

Relaxation energy deposition is simply the negative first moment of the Auger production feed function (Eq. 14).

20 $$\begin{aligned} {{\mathscr {E}}}_{g'}^{a}(E_g) = - {{\mathscr {F}}}_{0g'}^{a,1}(E_g). \end{aligned}$$

Charge deposition cross sections account for single electron reckoning, i.e., its deposition and removal, from the interaction site. They are therefore obtained in the same manner as to Eqs. 18–20 depositions, except with zero moments of the feed functions. Charge deposition feed functions are, by definition, associated with non-zero absorption interactions, and are given by:

21 $$\begin{aligned} {{\mathscr {C}}}_{g'}^{c}(E_g)= & {} \sum _k {{\mathscr {T}}}_{g'}^k(E_g) - \sum _{h=1}^{g'-2} {{\mathscr {F}}}^{k,ee,0}_{0 h}(E_g) + {\mathscr {F}}^{k,e\delta ,0}_{0 h}(E_g) \end{aligned}$$

22 $$\begin{aligned} {\mathscr {C}}_{g'}^{b}(E_g)= & {} {{\mathscr {T}}}_{g'}^b(E_g) - \sum _{h=1}^{g'-2} {\mathscr {F}}^{b,0}_{0 h}(E_g) \end{aligned}$$

23 $$\begin{aligned} {{\mathscr {C}}}_{g'}^{a}(E_g) = - {{\mathscr {F}}}_{0g'}^{a,0}(E_g). \end{aligned}$$

Both ${{\mathscr {E}}}_{g'}^{x}$ and ${{\mathscr {C}}}_{g'}^{x}$ concepts were introduced by Lorence et al.^66^, used by Acuros for within patient dose distribution and damage quantification^53^ and are generalized here for the ELECTR module.

### Dose deposition

The dose deposited is calculated in the Dragon-5 solver. Two quantities are needed: (1) the multigroup flux distribution ($\Phi _g$) obtained in Dragon-5 after solving Eq. 1 and integrating over all directions; and (2) the energy deposition cross sections provided by ELECTR (Eqs. 18–20). Let *T* be the irradiation time and $\rho$ the voxel density, the total dose distribution (in Gy) is given by:

24 $$\begin{aligned} D_t(\vec {r}) =\frac{T}{\rho (\vec {r})} \left[ \sum _x \sum \limits _{g}^{N_i} \langle E \rangle _g \Sigma _{t,g}^x(\vec {r}) \Phi _g(\vec {r}) - E_g \sum _{k=1}^{g} \Sigma _{0,k\rightarrow g}^{x} \Phi _k(\vec {r}) + \beta _g(\vec {r}) \Phi _g(\vec {r}) + \beta _N \Phi _N \right] . \end{aligned}$$

where $E_g$ refer to the midpoint energy for group *g* and $\langle E \rangle _g$ to the flux-weighted average energy for group *g*. Eq. 24 shows three components: (1) catastrophic energy deposition; (2) continuous slowing down ($\beta _g \Phi _g$) and 3) energy deposition below the cutoff ($\beta _N \Phi _N$). If an electron reaches an energy below ${1}\,{\textrm{keV}}$, it is stopped and its energy is deposited locally. As explained by Morel et al.^99^, by manipulating Eq. 24 and using Eqs. 18–20, the dose deposited is simplified as follows:

25 $$\begin{aligned} D_t(\vec {r})=\frac{T}{\rho (\vec {r})} {\sum \limits _{g}^{N_i}} \left[ {{\mathscr {E}}}_{g'}^{c}(E_g) + {{\mathscr {E}}}_{g'}^{b}(E_g) + {{\mathscr {E}}}_{g'}^{a}(E_g) \right] \Phi _g(\vec {r}) + \beta _N \Phi _N. \end{aligned}$$

### Algorithm coding and computational scheme

A pointwise-ENDF (PENDF) file, which contains electron cross sections on a unionized and linearized energy grid, is first generated using the NJOY RECONR module. A simplified representation of the ELECTR algorithm’s core is shown in Algorithm 1. ELECTR first reads the user’s input. The desired material (MAT) is then located on the input PENDF and stopping power ESTOP tapes. Stopping powers are not provided in the EPICS-2017 data. A CONVER module in NJOY has been developed to convert total stopping powers from CEPXS (Tape 9) into ENDF-6 format readable by ELECTR. For every atom’s subshell, the erelax subroutine stores the binding energy, the transition type (radiative or not), its efficiency and the emitted particle energy. Fluorescences, Augers, Coster–Kronig and super Coster–Kronig destination groups are then fixed in accordance with the user group structure.
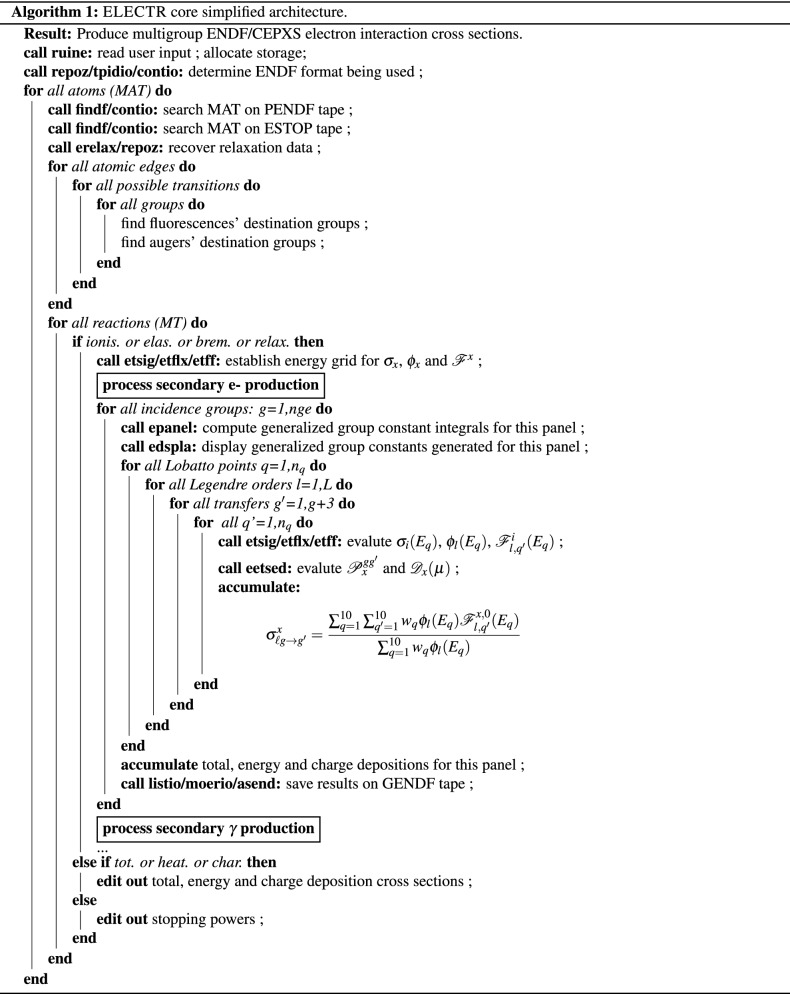


For each interaction process, the NJOY GROUPR module panel logic is adapted to average microscopic catastrophic cross sections (Eq. 6). Since each integrand in Eq. 6 has its own characteristics, the first calls and operations aim to unify the integration grid and quadrature schemes and detect discontinuities. The etsig subroutine is called to interpolate PENDF $\sigma _x$ at $E_g$ incidence energy and returns the next grid energy. The etflx and eetff subroutines perform similar operations for the electron flux and feed functions. The subroutine eetsed is called in both eetff and epanel cores. In the first call, scratch storage is allocated and all the ENDF subsections are read in. In the second call, angular and energy distribution kernels (${{\mathscr {P}}}_x$ and ${{\mathscr {D}}}_x$ in Eq. 8) are interpolated using the unit-base interpolation technique. A union grid is then generated for the three integrands $\sigma _x$, $\phi _x$ and ${{\mathscr {F}}}^x$. The integration algorithm is equivalent to the Minx legacy adaptative algorithm^100^.

Group-to-group catastrophic transfer matrices are computed for all secondary energy groups and all Legendre orders simultaneously. After all reactions are processed for this atom, a special pass is called to compute and store total catastrophic cross sections, total energy and charge deposition cross sections, as well as total restricted stopping powers. The NJOY upgraded MATXSR post-processing module formats the generated GENDF library in a single stand-alone multigroup dataset that can be accessed by a variety of legacy discrete ordinates and lattice codes. The MATXS library is used in conjunction with the Dragon-5 BFP solver^67^. Our deterministic computational scheme goes as follows. Depending on the mixture density and composition, microscopic cross sections are extracted, prepared and converted to macroscopic ones, respectively, by the $\texttt {LIB:}$ and $\texttt {MAC:}$ modules. Medium self-polarization reduces collisional stopping power. The latter appears in both restricted stopping power (Eq. 16) and energy deposition cross section (Eq. 18). A Sternheimer density effect correction^101,102^ is implemented and applied in Dragon-5 on both quantities (STERN 1). Region mixtures with vacuum boundary conditions are defined by the GEO: module. Discrete ordinate integration lines, region identification pointers and all tracking information are generated by the SNT: module. A High-Order Diamond Differencing (HODD) is used for the spatial discretization with a linear Legendre spatial order^103^. A convergence criterion of $1\times 10^{-5}$ was imposed on the flux inner iterations. The PSOUR: module is called to compute Eq. 1 right-hand-side source term. Since we limited our study to pure electron transport, PSOUR: will not compute corresponding bremsstrahlung and fluorescence photon sources. The ASM: assembly module recovers tracking lengths and material numbers from previous sequential tracking and computes BFP group-dependent system matrices. The linear BFP multigroup equation is finally solved by the FLU: module. Multigroup electron flux and macroscopic energy deposition cross section are used to compute dose spatial distributions by the HEAT: module.

## Results

**Figure 1:**
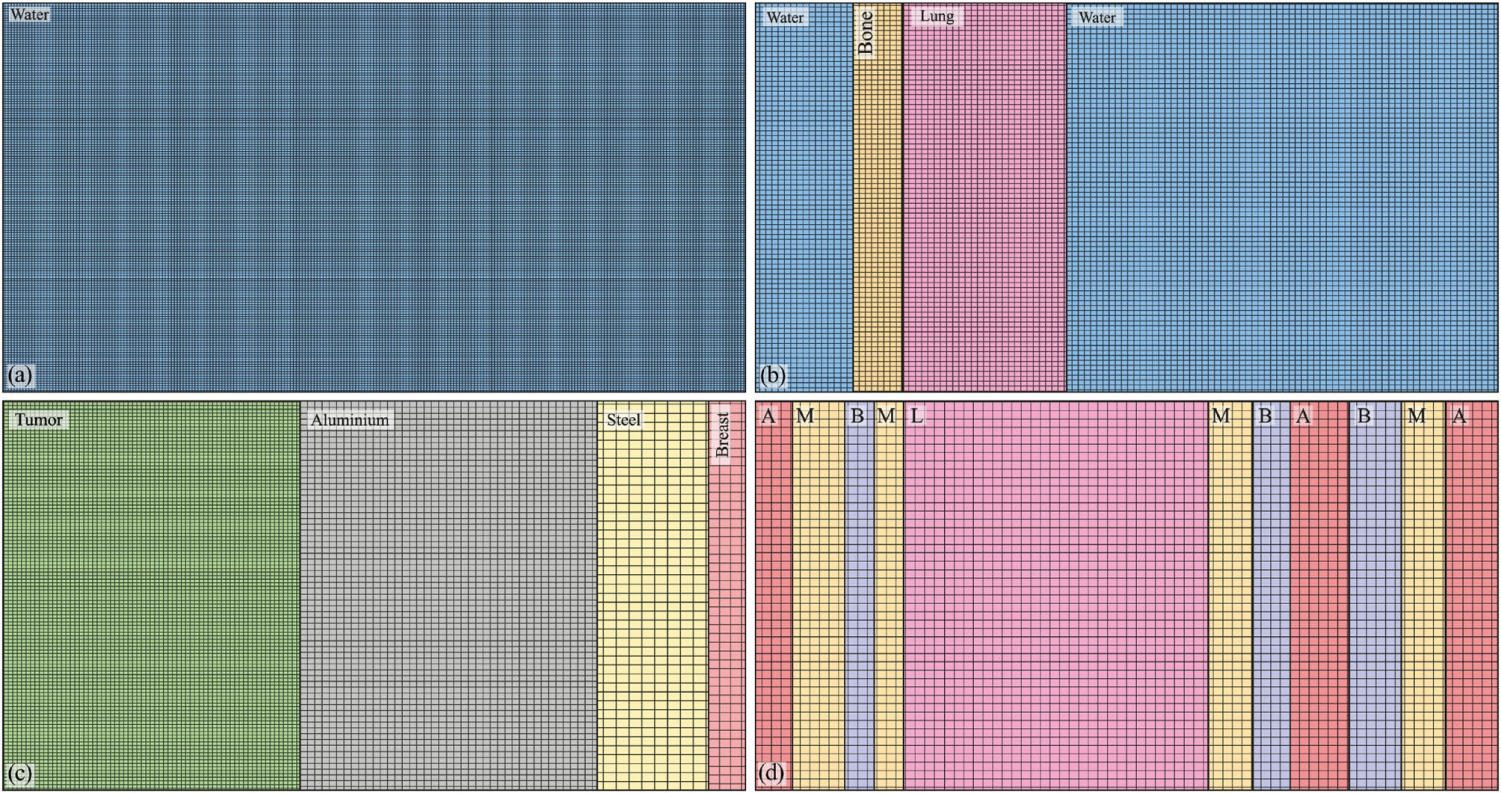
Benchmarks geometry: (**a**) water benchmark; (**b**) thorax benchmark; (**c**) intra-operative surgical benchmark and (**d**) high-heterogeneity benchmark. The material compositions are those of NIST. The electron source is monoenergetic and unidirectional. Lateral dimensions and source dimensions are fixed to the incident beam’s range. The percentage of materials’ thicknesses is then deduced from these dimensions. Longitudinal dimensions are infinite.

### Zero to medium heterogeneity benchmarks

We propose a validation of the NJOY-Dragon chain with a set of an increasing complexity benchmarks. Furthermore, a gradual increase in the beam energy is considered to challenge Legendre anisotropy, $S_{N}$ order, deterministic transport correction and number of energy groups. In the category of zero to medium heterogeneity benchmarks, Fig. 1a–c illustrates the case studies to be considered, where a unidirectional electron beam irradiates a homogeneous water (W) slab, a thorax (T) benchmark (water [$13\%$], bone [$7\%$], lung [$22\%$] and water [$58\%$]) and a breast intra-operative electron radiation therapy (IORT) benchmark (tumor [$40\%$], Al [$40\%$], steel [$15\%$] and tissue [$5\%$]). The benchmarks’ lateral dimensions are fixed to the incident beam’s range. The percentage of materials’ thicknesses is then deduced from these dimensions. The longitudinal dimensions in Geant-4 are infinite. The BFP equation in Dragon-5 is solved in 1D. This strategy makes it possible to study the net performance of the multigroup library on the depth dose profile without there being an accumulation of numerical errors related to the resolution of Eq. 1 in 2D and 3D. We thus avoid ray effects, an exorbitant angular discretization of the transport kernel and negative consequences from the high dimensionality of the Legendre scattering matrices. The use of unidirectional beams increases the computational complexity as well as the CPU time for two reasons. The first is that more focused interactions per unit length are imposed.

**Figure 2:**
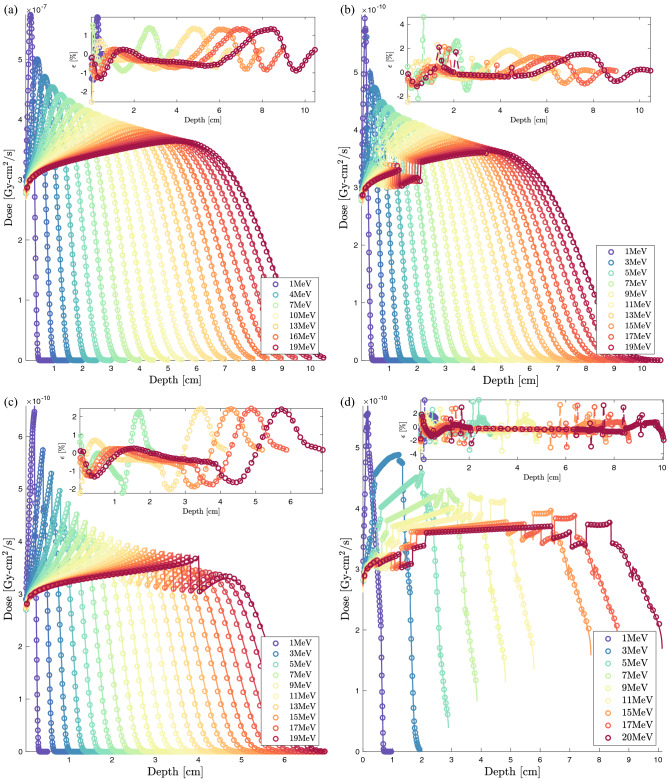
NJOY-Dragon-5 depth-dose curves (*solid lines*) compared to Geant-4 (*circles*) for 1 to ${20}\,{\textrm{MeV}}$ unidirectional electron beams incident on: (**a**) water benchmark; (**b**) thorax benchmark; (**c**) IORT-benchmark and (**d**) high-heterogeneity benchmark. Insert shows Dragon-5 relative error with respect to Geant-4. Normalization of any relative error presented is made with respect to the maximum dose observed for the incident beam. Monte Carlo convergences are obtained for a $0.2\%$ mean standard deviation.

The second is that the counterbalancing effects of dose errors at the interfaces and at the buildup for isotropic incidences are avoided. The W-benchmark is a rudimentary case study, which remains of clinical interest for both daily calibration verification and treatment planning. The T-benchmark introduces a first level of complexity with high- and low-density thin heterogeneities. The latter brings out the interface effects. The IORT-benchmark introduces a second level of within patient complexity combining internal (tissues) and external (instrumental) heterogeneities. High *Z* heterogeneities highlight shielding effect before a typical OAR. Incident beam energies vary from 1 to ${20}\,{\textrm{MeV}}$, covering the entire energy spectrum of clinical and medical research accelerators^104^. This also covers the entire electron radiotherapy and radiosurgery clinical beams’ spectra. Although electron beam treatments represent only 10–15% of daily workload in clinical practices^14^ and are constantly declining with the development of IMRT^105^, developing pure electron transport capabilities remains the preliminary step before fully-coupled [$\gamma ,\text {e}^-,\text {e}^+$] transport for photon beam treatments.

**Figure 3:**
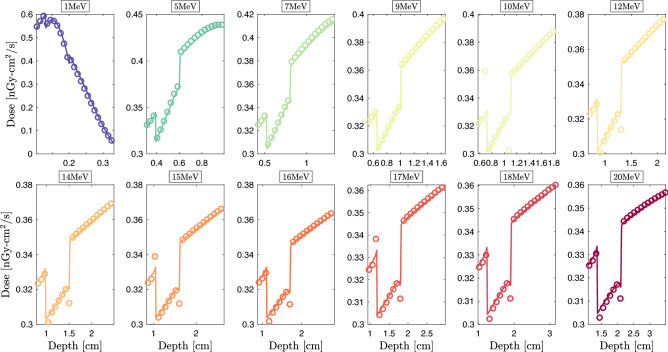
Njoy-Dragon-5 depth-dose curves (*solid lines*) compared to Geant-4 [G4EmLivermore (*circles*)] for incident unidirectional electron beams around the T-bone’s slab. Showed slabs include from the *left* to the *right*: water, bone and lung tissue. Dimensions are fixed to the incident beam’s range. MC convergences are obtained for a $0.2\%$ mean standard deviation.

Here, our goals are to: (1) demonstrate the accuracy of the ELECTR module, and (2) highlight, if any, the limitations of the CEPXS feed functions. As an immediate consequence, reducing the CPU time is not of interest in this study. For this reason, further optimizations of deterministic parameters ($N_{g},P_l,S_N,{{\mathscr {D}}}_r$) are not discussed here, where ${{\mathscr {D}}}_r$ refers to the discretization of the spatial domain. Conservative parameters are selected to eliminate deterministic bias when evaluating the accuracy of the ELECTR module. All computational schemes therefore relied on $N_g=300$ groups, $S_{48}$ order and ${{\mathscr {D}}}_r\ge 500$ elements. Legendre anisotropy is fixed to $P_9$ for 1 to ${6}\,{\textrm{MeV}}$, $P_{10}$ for 7 to ${9}\,{\textrm{MeV}}$, $P_{11}$ for 10 to ${14}\,{\textrm{MeV}}$ and $P_{12}$ for 15 to ${20}\,{\textrm{MeV}}$ electron beams. Convergence calculations were performed to support such choices. Only electrons are transported. Incident beams are assumed clean without any contamination.

For the Geant-4 reference scheme,$15\times 10^{6}$ primary events are considered for a $0.2\%$ statistical uncertainty (or better) in every material. Unless otherwise specified, we consider the physics constructor G4EmLivermore based on EPICS data (EADL^94^, EEDL^83^ and EPDL^95^). To be consistent with Dragon-5, transport cutoff and secondary production threshold are both fixed to ${1}\,{\textrm{keV}}$. The identity of each secondary particle is checked at each step of the track. The fluorescence and bremsstrahlung photons are thus eliminated at the point of birth. Figure 2a shows Dragon-5 versus Geant-4 depth-dose curves for the W-benchmark (Fig. 1a). As the beam’s energy increases, build-up spreads and the Legendre requirements are therefore higher. We observe that the buildup inflection dictates the $P_l$ order requirement. This can be explained by Mott and Møller’s increasing anisotropy with the beam energy. The insert shows the relative BFP deviation with respect to the MC scheme. This was obtained, in post-processing, following a piecewise cubic Hermite grid unification of Geant-4 and Dragon-5 dose detectors. The average BFP-MC error (in absolute value) is of 0.43$\%$, 0.58$\%$, 0.52$\%$ and 0.53$\%$, respectively, at 1, 9, 15 and 20 MeV. This average is on the entire spatial domain. Table 1 shows voxels’ percentage satisfying the $2\%$ criterion. For all beams examined, 100$\%$ of the water voxels satisfy this criterion. This agreement is reduced to 85$\%$ for a 1$\%$ BFP-MC deviation criterion. Figure 2b shows Dragon-5 vs. Geant-4 depth-dose curves for the T-benchmark (Fig. 1b). The Legendre anisotropy increase with energy is true for all benchmarks. At ${9}\,{\textrm{MeV}}$, the mean BFP-MC error is 0.76$\%$, 0.53$\%$, 0.68$\%$ and 0.50$\%$, respectively, in the first water slab, bone, lung and the last slab of water. For the same materials, these deviations are of the order of 0.62$\%$ (0.62$\%$), 0.93$\%$ (0.80$\%$), 0.48$\%$ (0.40$\%$) and 0.57$\%$ (0.62$\%$) for the ${15}\,{\textrm{MeV}}$ (${20}\,{\textrm{MeV}}$) beam. From Table 1, 98.2$\%$, 99.2$\%$, 98.4$\%$ and 99.4$\%$ of the thorax voxels satisfy the $2\%$ criterion, respectively, at 1, 9, 15 and ${20}\,{\textrm{MeV}}$. At ${1}\,{\textrm{MeV}}$, there is a loss of precision in lung voxels compared to higher energy beams. The slight loss of BFP-MC agreement, in some voxels, is caused by the bone interface. If one disregard the bone interface discontinuity, 99.8$\%$ of the thorax voxels satisfy the $2\%$ criterion (all beams combined). Figure 3 points out that the way Geant-4 handles this discontinuity is ambiguous. We tested other physics list constructors, namely G4EmPenelope, G4EmLowPhysics, G4EmStandard and G4EmStandard_opt4. The same failures, as those of G4EmLivermore (Fig. 3), persist. This Geant-4 failure can be corrected either (1) by forcing the electron step size not to exceed $25\%$ of the voxel’s thickness; or (2) by optimizing control constants of the G4UrbanMscModel condensed-history (CH) algorithm^106^. The Geant-4 team advises option 1. Although more physical, the option 2 is very time-consuming^107^. Finally, the post-processing piecewise cubic Hermite interpolation of the Geant-4 dose for a unique voxel grid MC-BFP comparison causes this single anomaly to propagate at intermediate voxels, thus further reducing the percentage at Table 1.

**Figure 4:**
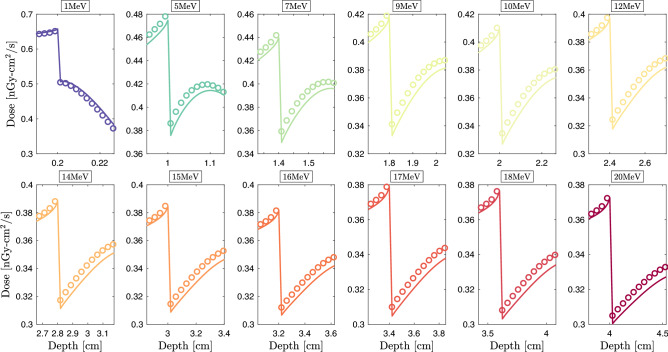
Njoy-Dragon-5 depth-dose curves (*solid lines*) compared to Geant-4 [G4EmLivermore (*circles*)] for incident unidirectional electron beams around the tumor [*left*] -aluminium [*right*] transition in the IORT-benchmark. Dimensions are fixed to the incident beam’s range. MC convergences are obtained for a $0.2\%$ mean standard deviation.

The same depth-dose curves for the IORT-benchmark (Fig. 1c) are depicted in Fig. 2c. Doses at the aluminum interfaces as well as within the tumor buildup are in close agreement between Dragon-5 and Geant-4. For the ${9}\,{\textrm{MeV}}$ beam, the average BFP-MC deviation is 0.60$\%$, 0.98$\%$, 0.00$\%$ and 0.00$\%$, respectively, in tumor tissue, aluminum, steel and breast tissue. These deviations are of 0.47$\%$ (0.46$\%$), 0.99$\%$ (0.92$\%$), 0.00$\%$ (0.00$\%$) and 0.00$\%$ (0.00$\%$) for the ${15}\,{\textrm{MeV}}$ (${20}\,{\textrm{MeV}}$) beam. Both Dragon-5 and Geant-4 electron doses are zero in the steel slab and breast tissue, which means that a complete attenuation of the beam was achieved in the aluminum slab for both codes. The T-bone interface anomaly (Fig. 3) disappears at the aluminum interface. The percentage of voxels satisfying the $2\%$ criterion is 99.27$\%$, 94.55$\%$, 96.00$\%$ and 96.36$\%$, respectively, for 1, 9, 15 and ${20}\,{\textrm{MeV}}$ beams. 99.7$\%$ of tumor and breast tissue voxels satisfy the $2\%$ criterion. Figure 4 confirms that the BFP-MC discrepancies are observed within $_{13}$Al buildup and not at the tumor-$_{13}$Al interface.

Using other physics list constructors will not fix this error. We will show that there is an acceptable match (less than $2\%$) between the dose predicted by Geant-4 and Dragon-5 in homogeneous $_{13}$Al slabs for all depths and beams. The $_{13}$Al buildup error can be fixed, as for the T-bone (Fig. 3), either by forcing tracking or refining the condensed history method. As soon as the particle enters a new volume or starts a new track, its step *s* is re-initialized according to $s=f_{r} \max (R,\lambda _1)$, where *R* is the electron range and $f_{r}$ ($\in [0,1]$) is a control constant. Here, all cross sections are assumed to be constant along *s*. It is advisable either (1) to restrict the value of *s* so that the stopping power does not vary by more than $20\%$ during the step^108^ or (2) to reduce *s* to less than $25\%$ of the detector’s width. Therefore, the public SetStepFunction of the base class G4VMultipleSacttering should be called for further electron step control. Two internal parameters need to be finely optimized: (1) dRoverRange which imposes a maximum step size given by the ratio step/range and (2) the finalRange. The MC electron transport then becomes extremely time-consuming; resolving this is beyond the scope of the current work.

**Table 1:** Percentage of voxels satisfying the AAPM $2\%$ criterion for a relative BFP-MC error below $2\%$. Listed values include buildup, interfaces and straggling regions. From top to bottom, the three showed sections are: the W-benchmark, the T-benchmark (and its materials) and the IORT-benchmark (and its materials).

Material	1 MeV	9 MeV	15 MeV	20 MeV
Water [1]	100.0	100.0	100.0	100.0
Water [2]	98.00	98.00	98.00	98.00
Bone [2]	82.35	92.16	88.24	94.12
Lung [2]	72.28	99.01	97.03	98.02
Water [2]	92.36	100.0	100.0	100.0
T-All [2]	98.20	99.20	98.40	99.40
Tumor [3]	99.50	99.50	99.50	99.50
Aluminium [3]	86.09	74.83	79.47	84.77
Steel [3]	100.0	100.0	100.0	100.0
Tissue [3]	100.0	100.0	100.0	100.0
IORT-All [3]	99.27	94.55	96.00	96.36

**Table 2:** Percentage of the HH-benchmark voxels satisfying the AAPM $2\%$ criterion for a relative BFP-MC error below $2\%$. Listed values include buildup, interfaces and straggling regions. From top to bottom, the materials listed are in their order of appearance from left to right in Fig. 1d.

Material	1 MeV	9 MeV	15 MeV	20 MeV
Adipose	100.00	92.00	99.00	91.00
Muscle	99.01	97.03	95.05	94.06
Bone	83.17	89.11	91.09	90.10
Muscle	92.08	93.07	92.08	91.09
Lung	99.01	99.01	99.01	99.01
Muscle	98.16	95.05	95.05	95.05
Bone	82.53	88.12	89.11	89.11
Adipose	98.57	94.06	95.05	95.05
Bone	100.0	94.06	93.07	93.07
Muscle	100.0	92.08	94.06	95.05
Adipose	100.0	94.17	95.39	96.33
All materials	99.73	97.12	98.34	97.02

Moreover, there is no clinical interest in being precise in the prediction of the dose deposition in the $_{13}$Al slab. However, the partial loss of precision in tumor tissue ($0.5\%$ of voxels, Table 1) is caused by the $_{13}$Al backscattering effect. The maximum error remains below $2.48\%$ (all voxels, beams and materials combined). One can question the validity of the ${1}\,{\textrm{keV}}$ transport cut-off imposed by the CEPXS data. The range of a ${1}\,{\textrm{keV}}$ electron in tissue is ${5}\,{\upmu {\textrm{m}}}$. Taking the worst-case scenario studied here, a ${1}\,{\textrm{MeV}}$ beam, typical for epithelial nonmelanoma skin cancer, will have a range of ${0.5}\,{\textrm{cm}}$. With the density of 500 voxels used in Dragon-5, this systematically results in a voxel having a ${10}\,{\upmu {\textrm{m}}}$ thickness; that is to say a voxel twice larger than the range of the electron.

### High-heterogeneity benchmark

Dragon-5 depth-dose curves compared to Geant-4 ones on the high-heterogeneity (HH) patient-like benchmark (Fig. 1d) are depicted in Fig. 2d. A good BFP-MC agreement is observed for any beam, material, interface and/or buildup. This benchmark adds a new level of complexity greater than the previous thorax and IORT case studies. Electron backscattering effects are more frequent, more intense and have a direct consequence on the dose. Unlike the T-benchmark (Fig. 3), the insert in Fig. 2d shows that interfaces’ discrepancies are much lower and we do not observe failures at the bone interfaces.

**Figure 5:**
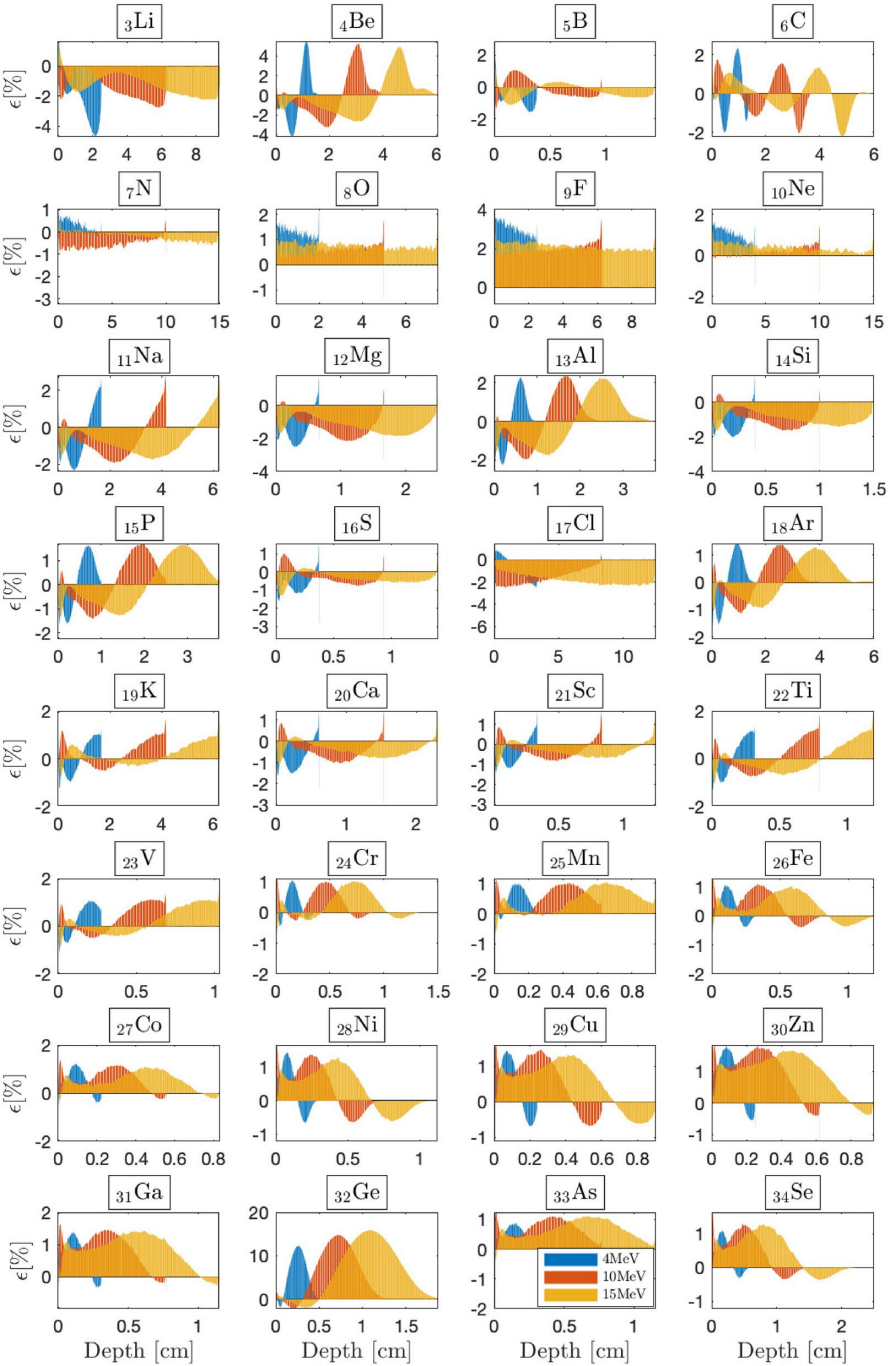
BFP-MC relative differences in energy deposition profiles versus depth for selected 4, 10 and ${15}\,{\textrm{MeV}}$ beams from $Z=3$ to $Z=34$. The irradiated slabs’ dimensions are fixed to the beam’s range within the irradiated material. Monte Carlo convergences are obtained for a $0.2\%$ mean standard deviation.

**Figure 6:**
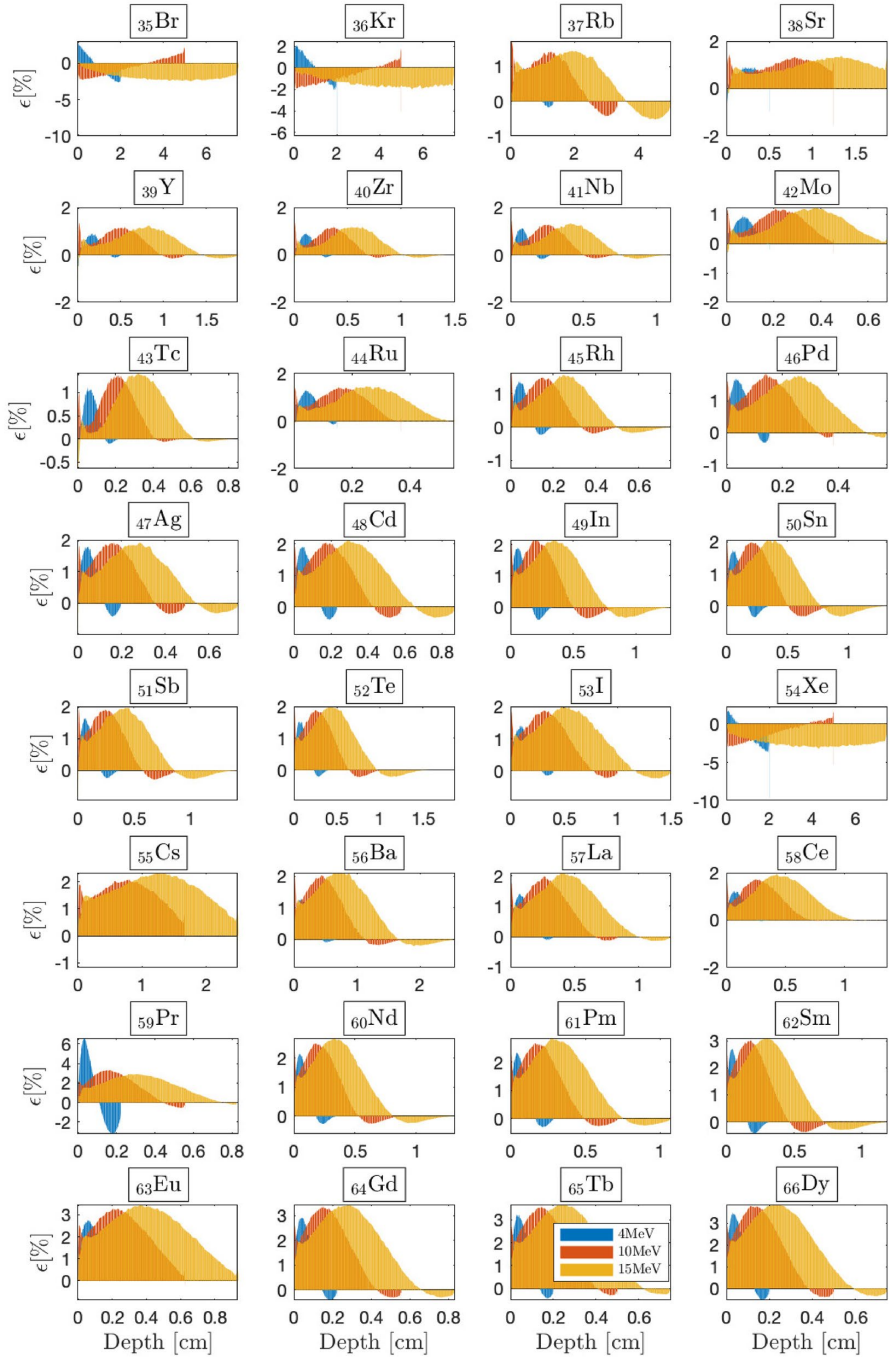
BFP-MC relative differences in energy deposition profiles versus depth for selected 4, 10 and 15 MeV beams from $Z=35$ to $Z=66$. The irradiated slabs’ dimensions are fixed to the beam’s range within the irradiated material. Monte Carlo convergences are obtained for a $0.2\%$ mean standard deviation.

**Figure 7:**
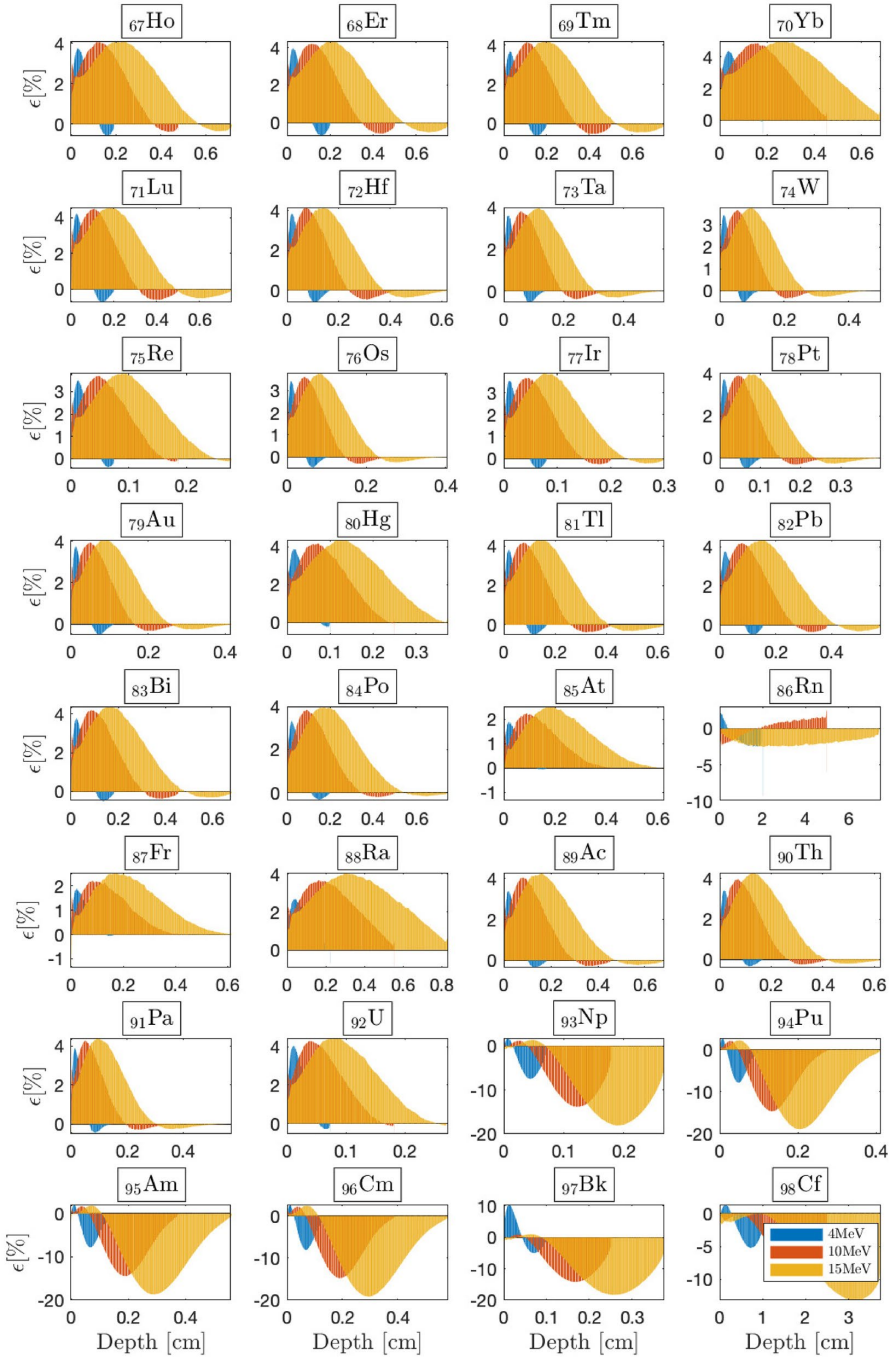
BFP-MC relative differences in energy deposition profiles vs. depth for selected 4, 10 and 15 MeV beams from $Z=67$ to $Z=98$. The irradiated slabs’ dimensions are fixed to the beam’s range within the irradiated material. Monte Carlo convergences are obtained for a $0.2\%$ mean standard deviation.

**Figure 8:**
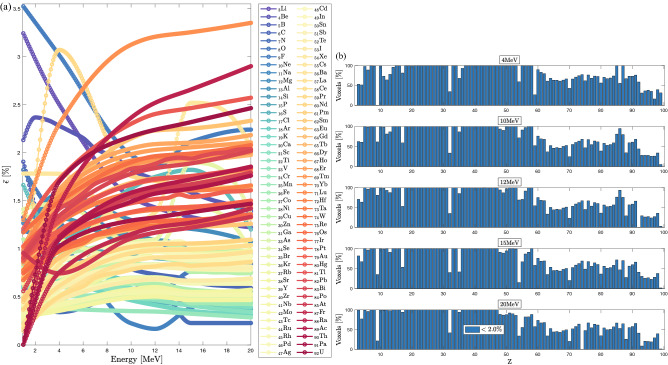
(**a**) Njoy-Dragon-5 mean relative errors in energy deposition profiles with respect to Geant-4 as a function of the incident beam’s energy. Curves are limited to Class-I and -II atoms. (**b**) Homogeneous atomic slabs’ voxels percentage satisfying a $2\%$ BFP-MC relative dose difference vs. *Z*. Monte Carlo convergences are obtained for a $0.2\%$ mean standard deviation.

This can be explained by errors’ counterbalancing from the neighboring slabs’ backscattering, given that we go from 4 (T) to 11 (HH) slabs. The average BFP-MC relative error is of $0.55\%$, $0.56\%$, $0.57\%$ and $0.57\%$, respectively, at 1, 9, 15 and ${20}\,{\textrm{MeV}}$. Table 2 shows the percentage of voxels satisfying the $2\%$ criterion for each of the 11 slabs. For the entire HH-benchmark, a minimum of $99.18\%$ of voxels satisfy the $2\%$ criterion, all beams combined. The percentage of the HH voxels that satisfy a limit of $1\%$ BFP-MC discrepancy is about $77.82\%$, $78.55\%$, $84.36\%$, $82.55\%$, respectively, for 1, 9, 15 and ${20}\,{\textrm{MeV}}$ electron beams.

### The limits of the CEPXS mode in ELECTR: a meta-analysis

Previous benchmarks are limited to the radiation oncology context. We now answer the question of what would be the limit of the CEPXS mode in ELECTR for beams from 1 to ${20}\,{\textrm{MeV}}$. We then propose an electron irradiation of successive homogeneous slabs covering the entire periodic table. The slabs’ dimensions are always fixed to the range of the incident beam. Transport remains restricted to electrons without considering the contamination effects. Figures 5, 6 and 7 show BFP-MC relative error in energy deposition profiles as a function of material depth from $Z=3$ (lithium) up to $Z=98$ (californium). Energy deposition is followed until complete beam attenuation is achieved. As a criterion for acceptable accuracy, we take a MC-BFP discrepancy below the threshold of $2\%$, i.e., in compliance with radiation oncology standards^14,50^. Apart from some exceptions, which we elaborate on in what follows, the criterion of $2\%$ is met for almost all voxels and beam energies, from $Z=3$ (lithium) to $Z=58$ (cerium) (Figs. 5, 6).

The range from $_3$Li to $_{58}$Ce forms a first class of CEPXS interest, which we denote Class-I. From $_{59}$Pr, we observe the emergence of a systematic MC-BFP deviation triggered around $D_{\text {max}}$, the depth of the maximum dose. Overall, the latter error both increases and expands with *Z*, independent of the beam energy. For the ${15}\,{\textrm{MeV}}$ beam, at $D_{\text {max}}$, it goes from $2.268\%$ to $2.296\%$, $3.080\%$, $3.381\%$, $3.392\%$ respectively, for $_{60}$Nd, $_{61}$Pm, $_{62}$Sm, $_{63}$Eu and $_{64}$Gd slabs (Fig. 6). It increases slowly from $_{59}$Pr to $_{92}$U, where it stagnates at around $4.62\%$ at $D_{\text {max}}$ (Fig. 7). The increase of the MC-BFP gap in spatial expansion around $D_{\text {max}}$ is $\sim 4\%$ (of the total voxels) per increase of *Z* from $_{59}$Pr to $_{92}$U. For this second class of interest from $_{59}$Pr to $_{92}$U, which we denote Class-II, the failure concerns only the region around $D_{\text {max}}$. The buildup and the tail region are spared. A third CEPXS class of interest from $_{93}$Np to $_{99}$Es, denoted Class-III, arises for which the BFP-MC divergence is total and considerable (Fig. 7). There are exceptions to this classification. First, BFP transport is ambiguous for gaseous slabs, e.g., $_{1}$H, $_{2}$He, $_{9}$F and $_{54}$Xe. Understanding the origin of this deterministic transport anomaly in gaseous media is beyond the scope of this study. The literature does not address this topic either. Second, $_{32}$Ge is an exception to the performance of Class-I materials (Fig. 5), i.e., the failure is total for this slab ($\sim 15\%$ at ${15}\,{\textrm{MeV}}$ around $D_{\text {max}}$). Third, the excellent performance for both $_{85}$At and $_{87}$Fr slabs is unexpected for Class-II atoms (Fig. 7). We therefore retain that the CEPXS feed functions in ELECTR are acceptable from $Z=1$ to $Z=58$, $_{85}$At and $_{87}$Fr, except for $_{32}$Ge, $_{3}$Li, $_{4}$Be and purely gaseous media.

Moreover, we observe that decreasing the densities of Class-II materials from those of NIST to ${1.0}{\mathrm{g/cm^3}}$ systematically reduces the BFP-MC error. This is because of an ensuing decrease in the interaction rate. For example, decreasing the $_{92}$U slab’s density from ${18.94}{\mathrm{g/cm^3}}$ to ${1.0}{\mathrm{g/cm^3}}$ reduces the maximum error from $4.62\%$ to $2.50\%$ at $D_{\text {max}}$. The error around $D_{\text {max}}$ is systematically corrected too. Therefore, Class-II materials can meet the $2\%$ criterion at the limit of a density reduction. However, the same test will not work for Class-III materials. For example, decreasing the $_{98}$Cf slab’s density (from 15.1 to ${1.0}{\mathrm{g/cm^3}}$) reduces the error from $22\%$ to just $12\%$ at $D_{\text {max}}$. While for Class-I and Class-II materials, the BFP-MC error is quite independent of the beam’s energy (Figs. 5, 6 and 7), Class-III materials’ errors are sensitive to the incident beam’s energy.

Figure 8a depicts BFP-MC error averaged over all voxels for Class-I and Class-II atoms as a function of the beam energy. However, the average error remains unrepresentative, given that in $95\%$ of cases, it remains below the $2\%$ criterion. On the other hand, Fig. 8a confirms that the MC-BFP deviation is (i) not very sensitive to the beam energy; and (ii) highly sensitive to *Z*. The distinction of Class effect is better identified in Fig. 8b, which shows the percentage of voxels with a BFP-MC relative error below $2.0\%$ as a function of *Z* for different beams. The transition from Class-I ($100\%$ of voxels) to Class-II ($\sim$30–80% of voxels) is obvious around $_{59}$Pr. The large increase in the error for Class-III and the decrease in the number of efficient voxels to the limit of $\sim 20\%$ is also evident, in Fig. 8b, around $_{93}$Np. The exceptions, in particular gas slabs and $_{32}$Ge from Class-I, $_{85}$At and $_{87}$Fr from Class-II, are noticeable in Fig. 8b.

### Experimental verification with Lockwood’s benchmarks

**Figure 9:**
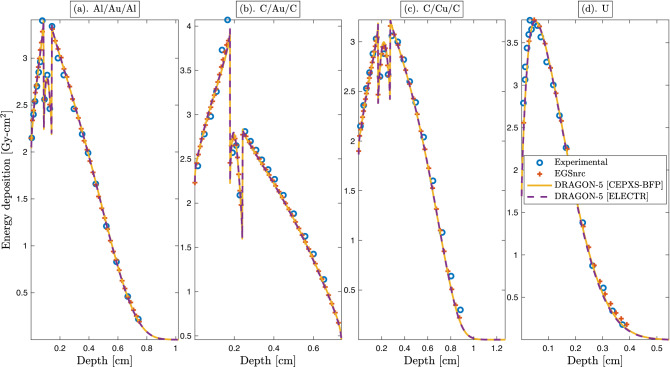
A 1 MeV unidirectional electron beam incident on: (**a**) Aluminium/Gold/Aluminium slabs; (**b**) Carbone /Gold/ Carbone slabs; (**c**) Carbone/Copper/Carbone; and (**d**) Uranium slab. Experimental energy depositions are from Lockwood’s calorimetric measurements^68^. Here, EGSnrc is the reference Monte Carlo code. Two deterministic calculations are shown. The first corresponds to Dragon-5 feeded with an FMAC-formatted library based on the CEPXS-BFP code, while the second to the NJOY [ELECTR]–Dragon-5 chain. Dimensions are fixed to the incident beam’s range within the irradiated benchmark.

Figure 9 shows energy deposition profiles of the BFP Dragon-5 solver vs. Lockwood’s experimental data^68^ and Egs-nrc. Two Dragon-5 computational schemes are examined; the first with the legacy CEPXS-BFP multigroup library^109^ and the second with the CEPXS mode in ELECTR. Lockwood’s calorimetric measurements are limited to 1 MeV unidirectional beams. The analyzed benchmarks are characterized by heterogeneous, high and medium *Z* materials. Four observations can be made. First, the CEPXS feed functions in ELECTR are equivalent to the CEPXS-BFP code. This provides strong evidence that ELECTR-calculated cross sections are reliable. Second, the Dragon-5-Lockwood difference is lower than the experimental data precision ($\sim 2\%$) for $98\%$ of the voxels (all benchmarks included). Third, the Dragon-Egs-nrc agreement is lower than the precision on the cross sections ($\sim 2\%$) for $100\%$ of the voxels. Fourth, there is an unexpected agreement for the uranium slab (Fig. 9d) given that this atom is of Class-II (Fig. 7). The uranium agreement may be explained by (1) the beam’s energy (in Fig. 9) which is very low for a clean classification; or (2) the G4EmLivermore library (in Figs. 5, 6, 7) could be problematic for this atom. Addressing this question would require an investigation beyond the scope of the current work.

## Conclusion

In this paper, we presented and validated ELECTR, a state-of-the-art multigroup electron cross section generation module in NJOY. ELECTR produces a GENDF-formatted library containing: multigroup total catastrophic electroatomic cross sections, relaxation cascade production, anisotropic Legendre components of within-group elastic and group-to-group catastrophic inelastic scattering matrices, radiative and collisional soft stopping powers, multigroup Fokker–Planck momentum transfer coefficients, energy and charge deposition cross sections. This validation was limited to the CEPXS mode in ELECTR. Therefore, the interactions handled include: Møller inelastic scattering, Mott elastic scattering, bremsstrahlung, but also fluorescence, Auger, Coster–Kronig and super Coster–Kronig electron productions. The NJOY MATXSR post-processing module was upgraded to format the produced GENDF library in a MATXS format. The Dragon-5 Boltzmann–Fokker–Planck (BFP) solver was also upgraded to access the MATXS library and solve the BFP equation. The validation of the Njoy-Dragon chain was proposed against experimental calorimetric data and Egs-nrc on Lockwood’s benchmarks, but also against Geant-4 on typical radiation oncology benchmarks. The limits of the CEPXS mode have been reported by irradiating slabs from $Z=1$ (hydrogen) up to $Z=99$ (einsteinium), from 1 to 20 MeV, covering the entire radiotherapy and radiosurgery clinical beams’ spectra.

For typical high and medium *Z* Lockwood’s slabs, Njoy-Dragon dose differences, with respect to calorimetric measurements, were below experimental precision for $99\%$ of voxels. Whereas for $100\%$ of voxels, Njoy-Dragon vs. Egs-nrc dose discrepancies were within the order of the precision on the cross sections. For homogeneous water slabs, for beams from 1 to 20 MeV, all voxels combined, the average BFP-MC error was below $0.52\%$. Of particular note, $100\%$ of water voxels satisfied the $2\%$ criterion. For the thorax benchmark, all beams combined, the average BFP-MC deviation was $0.65\%$, $0.75\%$, $0.52\%$ and $0.56\%$, respectively, in the first water slab, bone, lung and the last slab of water. Precisely, 99.2$\%$ and 98.4$\%$ of the thorax voxels satisfied the $2\%$ criterion, respectively, at 9 and 15 MeV. A systematic BFP-MC deviation was observed at the bone interface discontinuity point. This error is due to a crossing boundary problem on the Geant-4 side which can be resolved by forcing the step size of the electron’s track. For a typical Intra-Operative Radiotherapy (IORT) benchmark, all beams combined, the average BFP-MC relative error was $0.51\%$, $0.96\%$, $0.00\%$ and $0.00\%$, respectively, in tumor tissue, aluminum, steel and breast tissue. For all beams, $99.7\%$ of IORT tumor and breast voxels satisfied the AAPM $2\%$ criterion. Finally, for the high-heterogeneity patient-like benchmark, all beams combined, the average BFP-MC relative error was about $0.56\%$, while, in the worst-case, $97.0\%$ of adipose, muscle, bone and lung voxels satisfied the $2\%$ criterion. By irradiating homogeneous slabs from $Z=1$ (hydrogen) to $Z=99$ (einsteinium), we have determined the limits of the CEPXS mode in ELECTR. Apart from gas exception, there is an excellent BFP-MC agreement (below $2\%$) from $_{1}$H to $_{58}$Ce. Then, from $_{59}$Pr, a systematic deviation appears around $D_{\text {max}}$ (the maximum dose deposition depth). This error increases and expands with Z, independently of the beam’s energy. The error’s gain in spatial expansion, around $D_{\text {max}}$, is of $\sim 4\%$ (of total voxels) per 1-unit of Z increase from $_{59}$Pr to $_{92}$U. The error goes from $2.27\%$ for $_{60}$Nd to $4.62\%$ for $_{92}$U. From $_{93}$Np, a last category is observed for which the CEPXS data are no longer reliable. All the benchmarks studied are in 1D to exclude any deterministic bias in the validation of the multigroup library.

We believe that additional precision and quality assurance can be achieved with ENDF feed functions, i.e., EEDL-2017, EPDL-2017 and EADL-2017 data. One natural next step is therefore to study the potential performance of the ELECTR module in the ENDF mode. Such a library is expected to correct reported anomalies, cover conventional radiotherapy and radiosurgery treatment planning but also the very high-energy radiotherapy modality for deep-seated tumors. The ENDF mode will make it possible to use the same data on both sides (BFP and MC), which opens the door to sensitivity analyzes and the explanation of the origin of the differences. This library is expected to cover a wide range of energies from ${100}{\textrm{eV}}$ to ${100}{\textrm{GeV}}$.

## Data Availability

The datasets used and analyzed during the current study are available from the corresponding author on reasonable request. The DRAGON-5 code and the MATXS-formatted libraries are available under the GNU Lesser General Public License (LGPL). The ENDF mode in the ELECTR code will also be made available under open source license by 2024.
